# Hospital and outpatient clinic utilization among older people in the 3-5 years following the initiation of continuing care: a longitudinal cohort study

**DOI:** 10.1186/1472-6963-11-136

**Published:** 2011-05-31

**Authors:** Anna Condelius, Ingalill R Hallberg, Ulf Jakobsson

**Affiliations:** 1 Department of Health Sciences, Faculty of Medicine, Lund University, Lund, Sweden; 2 Center for Primary Health Care Research, Faculty of Medicine, Lund University, Malmö, Sweden

## Abstract

**Background:**

Few studies have investigated the subsequent rate of hospital and outpatient clinic utilization in those who receive continuing care and have documented frequent usage over one year. Such knowledge may be helpful in identifying those who would benefit from preventive interventions. The aim of this study was to investigate and compare the subsequent rate of hospital and outpatient clinic utilization among older people with 0, 1, 2, 3 or more hospital stays in the first year following the initiation of continuing care. A further aim was to compare these groups regarding demographic data, health complaints, functional and cognitive ability, informal care and mortality.

**Methods:**

A total of 1079 people, aged 65 years or older, who received a decision regarding the initiation of continuing care during the years 2001, 2002 or 2003 were investigated. Four groups were created based on whether they had 0, 1, 2 or ≥3 hospital stays in the first year following the initiation of continuing care and were investigated regarding the rate of hospital and outpatient clinic utilization in the subsequent 3-5 years.

**Results:**

Fifty seven percent of the sample had no hospital stay during the first year following the initiation of continuing care, 20% had 1 stay, 10% had 2 stays and 13% had three or more hospital stays (range: 3-13). Those with ≥3 hospital stays in the first year continued to have the significantly highest rate of hospital and outpatient care utilization in the subsequent years. This group accounted for 57% of hospital stays in the first year, 27% in the second year and 18% in the third year. In this group the risk of having ≥3 hospital stays in the second year was 27% and 12% in the third year.

**Conclusions:**

There is a clear need for interventions targeted on prevention of frequent hospital and outpatient clinic utilization among those who are high users of hospital care in the first year after the initiation of continuing care. Perhaps an increased availability of medically skilled staff in the day to day care of these people in the municipalities could prevent frequent hospital and outpatient clinic utilization, especially hospital readmissions.

## Background

Little is known about the subsequent rate of hospital and outpatient clinic utilization in people aged 65 years or above who have a history of frequent utilization of hospital care after receiving continuing care. It has been found that 18% of those who receive continuing care are high users of hospital care (≥3 admissions) and of outpatient care during one year [1]. Frequent hospital and outpatient clinic utilization may indicate an insufficient capacity within continuing care and home nursing care to provide care in such a way that deterioration in health and the need for such care is prevented. However, to justify interventions targeted on preventing frequent hospital and outpatient clinic utilization it is necessary to know how the utilization progresses in the time following the initiation of continuing care.

Care for frail older people in Sweden, and other countries as well [2], can be characterized by fragmentation with no one taking overall responsibility. The provision of hospital and outpatient care in Sweden rests with the county councils while the provision of continuing care and home nursing care rests with the municipalities [3]. Continuing care can be provided in the older person's home or in special accommodation (equivalent to a nursing home - housing with access to around-the-clock care and service) [4] after a needs assessment and a decision about how care is to be provided. It includes such tasks as help with laundry, cooking, cleaning, shopping and personal care, while home nursing care implies the provision of medical healthcare up to the level of registered nurses. Physicians are employed by the county councils i.e. in hospitals or in outpatient care [3] and consequently are available to the municipalities only as consultants. The proportion of people aged 65 years or older in Sweden who received continuing care and services from the municipality at home increased from 7% to 9% and the proportion in special accommodation decreased from 8% to 6% between the years 2000 and 2005 [5]. Continuing care is often carried out by assistant nurses or nursing aids whose medical competence tends to be low and work strain sometimes intolerably high [6]. High levels of work strain among district nurses providing home nursing care in the municipalities, in combination with the low formal competence among co-workers and the absence of physicians close at hand, have been shown to contribute to hasty decisions about referrals to hospital [7]. Thus, there may be factors in the way continuing care and home nursing care are provided to older people within the municipalities that might contribute to the demand for and use of hospital care. However, continuous contacts with primary care physicians have been shown to be of great importance in reducing the risk of readmission to hospital among older people [8,9]. Thus, utilization of hospital and outpatient care need to be investigated together.

Few studies have examined the subsequent rate of hospital and outpatient clinic utilization in those with documented frequent usage during one year, especially in those who receive continuing care. Roland et al. [10] investigated the pattern of emergency admissions during five years in a cohort of people aged ≥65, ≥75 and ≥85 years and with 2 or more admissions. This cohort was shown to have a 3.5 times higher readmission rate in the second year compared to the general population of the same age although the admission rate decreased in the following years. Roos, Shapiro and Tate [11] studied healthcare usage patterns over a 16-year period (1970-1985) in a randomly selected sample in Manitoba, Canada (n = 4209, age 65+). Their study showed that a minority of the population (5%) accounted for a majority of healthcare expenditure (68%) in the first year. The use of hospital care in one year was the best predictor of the next years´ usage but a less good predictor for usage in subsequent years. The longer the time that passed the less healthcare expenditure was centred on one group. Thus, utilization of hospital care seems to decrease over time among high users, even without targeted interventions. However, none of these studies have focused on those who receive continuing care or have investigated the utilization of outpatient care in parallel with hospital care utilization.

The aim of this study was to investigate and compare the subsequent rate of hospital and outpatient clinic utilization among older people with 0, 1, 2, 3 or more hospital stays in the first year following a decision to initiate continuing care. A further aim was to compare these groups regarding demographic data, health complaints, functional and cognitive ability, informal care and mortality.

## Methods

### Sample

The sample comprises people aged 65 years or older who received a decision to initiate continuing care in the years 2001, 2002 or 2003. The sample was drawn from the care and service part of the Good Ageing in Skåne Study (GAS) [12]. The inclusion criteria in GAS are: being 65 years or older; receiving continuing care and services from the municipality at home or in special accommodation; or receiving at least four home nursing and/or rehabilitation visits per month. All those who met the inclusion criteria were included and those who only received safety alarms, meals on wheels or transport services were excluded. Informed consent was obtained from all participants. GAS is a sub-study in the Swedish National Study on Ageing and Care (SNAC) [13] and proceeds in five municipalities in the region of Skåne in southern Sweden since 2001. The municipalities included represent both rural and urban areas.

### Data collection

Information about age, gender, functional ability, cognitive ability, health complaints, the date of the decision concerning the provision of continuing care and whether care was provided at home or in special accommodation at this point in time was collected through the care and services part of the GAS Study. Data were collected by means of a form completed by staff involved in care and services to older people in the municipalities (home help officers, registered nurses, physiotherapists or occupational therapists). The form is filled in when the decision concerning continuing care is made and then again every 6^th ^months or when there is change in the care the older person receives. The form was developed by an expert group and tested in a pilot study [14].

Data on medical healthcare utilization were derived from the Patient Administrative Support in Skåne (PASiS) and PrivaStat registers. PASiS is an administrative register of care and treatment provided by the Skåne County Council while PrivaStat is a register of care provided by private agencies in this region. The registers include information about admission and discharge dates from hospital and contact dates with the physician in outpatient care on an individual basis. The number of hospital stays and the number of contacts with the physician in outpatient care have been counted for each participant up to the year 2006. Thus, hospital and outpatient clinic utilization was followed for 5 years in the cohort included in 2001, 4 years in the 2002 cohort and 3 years in the 2003 cohort. GAS data were merged with data from PASiS and PrivaStat registers based on civil registration numbers. Data for death were collected from the Swedish population register.

The Ethics Committee at Lund University approved the study (LU 744-00, LU 650-00).

### Measurements

The ADL staircase [15] is included in the GAS form and was used in this study to measure performance in personal and instrumental activities of daily living (PADL/IADL). PADL includes: bathing, dressing, going to the toilet, transferring, continence and feeding; and IADL performance includes; cooking, transportation, cleaning and shopping. The performance in each activity is graded as dependent, partly dependent or independent. In this study the number of activities in which the person is graded as dependent in each section of the staircase has been calculated for each individual resulting in two sums: IADL sum ranging from 0- independent in all activities to 4- dependent in all activities and PADL sum ranging from 0- independent in all activities to 6- dependent in all activities.

The Berger scale [16] is also included in the GAS form and used in this study to assess cognitive ability. The scale has six levels. The classifications are: I "can function in any surroundings, but forgetfulness is often disruptive of daily activities"; II "can function without direction only in familiar surroundings"; III "needs direction to function even in familiar surroundings but can respond appropriately to instructions"; IV "needs assistance to function, cannot respond to directions alone"; V "remains ambulatory, needs assistance to function, but cannot communicate verbally in a meaningful fashion"; and VI "bedridden or confined to a chair and responds only to tactile stimuli" [16].

Health complaints were assessed using single items concerning dizziness, anxiety, depressed mood, pain and ulcers. For the questions concerning slow-healing wounds or pressure ulcers the response alternatives were "have ulcers" and "have no ulcers" while the response alternatives for the other health complaints were "periodic/slight", "periodic severe" and "constant severe". The questions regarding informal care in IADL or PADL have yes/no response alternatives.

### Statistical analysis

Four groups were created based on whether they had 0, 1, 2, 3 or more hospital stays in the first year following the decision about continuing care. These were then compared regarding demographic data, functional ability, cognitive ability, health complaints the number of hospital stays and the number of contacts with physicians in outpatient care and regarding the proportion who died 1-3 years following inclusion. It should be noted that the proportion who died each year was calculated based on the proportion still alive in the preceding year and not on the origin sample size. Comparisons were performed using chi-square tests for nominal data, the Kruskal- Wallis test or Mann-Whitney U-test for ordinal data and the ANOVA test or Student's t-test for numeric data. Changes in the mean number of hospital stays and in the mean number of contacts with physicians in outpatient care in the four groups, in the 3 years following inclusion, was investigated through repeated measure analyses. To achieve valid F-ratios in the analyses, Huynh-Felt's correction of the degree of freedom was used (when the assumption of sphericity was violated). Those for whom values were missing in any year under study (i.e. those who died during the study period) were automatically excluded from the repeated measure analyses. Thus, only those who remained alive throughout the study period were included in these analyses. Comparisons between those included in the repeated measure analyses and those who died were performed regarding the number of hospital stays and the number of contacts with physicians in outpatient care in the 2 years following inclusion. This was done using Student's t-test. A p-value of below 0.05 was regarded as significant except in the post-hoc analyses where a reduced p-value was used, in accordance with the Bonferroni method [17], to avoid mass significance. All analyses were performed using SPSS 15.0 for Windows.

## Results

A total of 1079 people were included in the study of whom 322 (30%) received a decision about continuing care during 2001, 411 (38%) during 2002 and 346 (32%) during 2003. At the time for inclusion the mean age in the total sample was 80.4 years (SD = 7.1), 64.5% were women and 37% received care in special accommodation. The mean IADL sum was 3.4 (SD 1.1) and 1.6 (SD 1.7) for PADL sum. The mean number of hospital stays in the first year was 0.9 (SD = 1.5). The mean number of contacts with physicians in outpatient care in the first year was 10.3 (SD = 9.3) ranging from 0 to 126. Two hundred and forty-seven people were not hospitalized during the three years under study and one person had not been in contact with a physician in outpatient care during this period. Forty-seven percent of the sample (511 people) had died by the end of the three years following inclusion. There were no significant (p = 0.05) differences between the cohorts regarding age (mean age in cohort I: 81.2 (SD = 7.4); cohort II: 80.3 (SD = 7.0); cohort III: 79.9 (SD = 7.9)) or in the proportion who died in the first (p = 0.9), second (p = 0.2) or third year (p = 0.9) following inclusion. Six hundred and twelve people (57%) had no hospital stay during the first year following the decision about continuing care, 217 (20%) had 1 stay, 110 (10%) had 2 stays and 140 people (13%) had 3 or more (range: 3-13).

### Comparisons of the groups with 0, 1, 2, or ≥3 hospital stays during the first year

Those with three or more hospital stays in the first year included a higher proportion of men, living at home, with no cognitive impairment and a higher proportion who suffered from pain and slow healing wounds, compared to those with one or no hospital stays (Table 1). These differences remained significant only between those with three or more hospital stays and those with none once post hoc analyses were performed. The mean number of hospital stays among those with three or more stays the first year remained above 1 in the following two years (mean second year: 1.9, mean third year: 1.1) which was significantly higher than in the other groups. Utilization of outpatient care differed significantly in all three years with those with 3 or more hospital stays in the first year having the highest number of contacts in all years. The mean number of contacts with physicians in outpatient care remained above 7 in all groups for all three years. Those with no hospital admissions in the first year received less informal care and had a significantly lower mortality rate the first and the second years compared to the other groups. There were no significant differences between groups regarding the proportion who died in the third year, in IADL, PADL, dizziness, anxiety, depressed mood or pressure ulcers (Table 1).

**Table 1:** Comparisons of the groups with 1, 2 or ≥3 hospital stays in the first year after the decision to initiate continuing care

	0 hospital stay *n *= 612		1 hospital stay *n *= 217		2 hospital stays *n *= 110		≥3 hospital stays *n *= 140		*p*-value	Post-hoc analyses^e^
Age										
Mean (SD)	81.0	(7.1)	79.9	(7.3)	80.0	(6.8)	78.9	(6.9)	0.010^a^	^-^
Sex										
Female %	69.8		59.9		58.2		54.3		< 0.001^b^	C ^b^
Living conditions %									< 0.001^b^	C ^b^
Home	59.6		67.5		71.6		76.6			
Special accommodation	40.4		32.5		28.4		23.4			
Dependent in IADL (%)										
Cleaning^f^	88.2		89.3		88.3		87.0		0.937^b^	
Shopping^f^	85.9		88.2		84.6		86.8		0.804^b^	
Transportation^h^	85.8		85.4		86.7		85.7		0.994^b^	
Cooking^f^	76.3		77.9		74.5		74.6		0.887^b^	
IADL sum Mean (SD)	3.4	(1.1)	3.4	(1.1)	3.4	(1.1)	3.5	(0.9)	0.922^a^	
Dependent in PADL (%)										
Bathing^f^	55.1		56.7		59.6		44.5		0.084^b^	
Dressing^f^	32.5		32.2		24.3		24.4		0.139^b^	
Toileting^f^	34.3		34.6		28.8		24.2		0.110^b^	
Transfer^f^	27.1		32.1		23.6		20.1		0.139^b^	
Continence^i^	44.5		44.7		47.2		40.4		0.801^b^	
Feeding^f^	3.9		3.4		2.9		0.8		0.327^b^	
PADL sum Mean (SD)	1.6	(1.7)	1.6	(1.7)	1.6	(1.6)	1.3	(1.6)	0.585^a^	
Cognitive ability ^f^									0.003^c^	C, E^d^
No impairment	53.8		59.0		61.8		75.2			
Severity class I	13.1		14.1		9.8		5.4			
Severity class II	9.7		8.3		10.8		5.4			
Severity class III	11.8		8.8		13.7		10.9			
Severity class IV	7.6		5.9		3.9		3.1			
Severity class V	1.6		2.0		.0		.0			
Severity class VI	2.4		2.0		.0		.0			
Dizziness (%) ^i^									0.930^c^	
Periodic dizziness	33.2		33.7		25.8		29.3			
Periodic severe dizziness	4.9		4.6		7.9		6.0			
Constant severe dizziness	0.8		2.3		3.4		4.3			
Anxiety (%) ^g^									0.101^c^	
Periodic anxiety	31.6		27.8		27.5		27.9			
Periodic severe anxiety	9.6		5.9		4.9		6.6			
Constant severe anxiety	1.2		2.0		1.0		1.6			
Depressed mood (%) ^g^									0.427^c^	
Periodic depressed	35.6		30.8		29.0		32.5			
Periodic severe depression	5.6		5.0		6.0		4.1			
Constant severe depression	1.1		1.5		.0		1.6			
Pain (%) ^h^									0.003^c^	C^d^
Slight pain	35.4		42.5		40.4		43.5			
Periodic severe pain	12.5		15.5		17.0		18.3			
Constant severe pain	2.6		3.3		3.2		4.3			
Ulcer (%)										
Slow- healing wounds^h^	4.2		7.5		4.3		12.8		0.003^b^	C^b^
Pressure ulcer ^h^	3.3		5.2		3.2		2.5		0.567^b^	
Number of hospital stays Mean (SD)										
Second year	0.6	(1.1)	0.8	(1.2)	0.9	(1.6)	1.9	(2.4)	< 0.001^a^	C, E, F
Third year	0.5	(1.1)	0.7	(1.6)	0.6	(1.1)	1.1	(2.4)	0.005^a^	C
Number of contacts with physicians in outpatient care Mean (SD)										
First year	8.1	(8.5)	11.5	(8.3)	12.2	(8.4)	16.5	(11.5)	< 0.001^a^	A, B, C, E, F
Second year	8.3	(8.5)	10.5	(9.4)	10.7	(7.4)	12.7	(11.3)	< 0.001^a^	A, C
Third year	7.0	(8.3)	9.6	(13.0)	7.9	(6.3)	12.9	(13.5)	< 0.001^a^	C, F
Deceased %										
First year	15.4		30.4		38.2		31.4		< 0.001^b^	A, B, C^b^
Second year	15.6		21.9		17.6		32.3		0.001^b^	C^b^
Third year	16.0		17.8		14.3		13.8		0.889^b^	
Informal care in IADL (%) ^g^	54.9		61.9		72.8		77.6		< 0.001^b^	B, C^b^
Informal care in PADL (%) ^g^	23.4		30.4		37.9		40.0		< 0.001^b^	B^b^

^a^) ANOVA ^b^) Chi square test ^c^) Kruskal-Wallis one-way analysis of variance test ^d^) Mann-Whitney U-test between two groups

Missing/unknown: ^f^4-7% ^g^7.1-10% ^h^11-15% ^i^16-18.2%

Reduced p-value < 0.012 was used in Post Hoc analyses according to the Bonferroni method

^e)^Significant differences between: (A) 0 hospital stay versus 1 hospital stay, (B) 0 hospital stay versus 2 hospital stays, (C) 0 hospital stay versus 3 hospital stays, (D) 1 hospital stay versus 2 hospital stays, (E) 1 hospital stay versus 3 hospital stays,(F) 2 hospital stays versus ≥3 hospital stays

Those with 3 or more hospital stays in the first year accounted for 57% of the total number of hospital stays and 21% of total number of contacts with physicians in outpatient care the first year (Table 2). In the third year this group accounted for 18% of the total number of hospital stays and 15% of contacts with physicians in outpatient care. The group with no hospital stays in the first year increased their proportion of the sample size (57% first year, 65% the third year), their proportion of the samples´ total number of hospital stays (0% first year, 53% the third year) and their proportion of the total number of contacts in outpatient care over the years (44% the first year, 56% third year) (Table 2). Of the group with no hospital stay in the first year, 7.0% had 3 or more hospital stays in the second year and 4.8% in the third year. In the group with one hospital stay in the first year, 8.6% had 3 or more stays the second year and 17.8% the third year. Among those with 2 hospital stays in the first year, 11.8% had 3 or more stays in the second year and 7.1% in the third year. In the group with 3 or more hospital stays in the first year 27.0% also had 3 or more stays in the second year and 12.3% the third year.

**Table 2:** The number of people, the number of hospital stays and the number of contacts with outpatient care in the 1-3 years following inclusion in the total sample and for each subgroup with 0, 1, 2 or ≥3 hospital stays in the first year, and the proportion accounted for by each subgroup

	Year 1			Year 2			Year 3		
	
	*N *people (% of total)	*N *hospital stays (% of total)	*N *contacts with physicians in outpatient care (% of total)	*N *people (% of total)	*N *hospital stays (% of total)	*N *contacts with physicians in outpatient care (% of total)	*N *people (% of total)	*N *hospital stays (% of total)	*N *contacts with physicians in outpatient care (% of total)
Total sample	1079 (100)	1014 (100)	10850 (100)	833 (100)	661 (100)	7771 (100)	676 (100)	380 (100)	5457 (100)
0 hospital stay	612 (56.7)	0 (0.0)	4763 (43.9)	518 (62.2)	302 (45.7)	4248 (54.7)	437 (64.6)	202 (53.2)	3041 (55.7)
1 hospital stay	217 (20.1)	217 (21.4)	2456 (22.6)	151 (18.1)	118 (17.8)	1578 (20.3)	118 (17.5)	75 (19.7)	1137 (20.8)
2 hospital stays	110 (10.2 )	220 (21.6)	1334 (12.3)	68 (8.2)	60 (9.1)	727 (9.3)	56 (8.3)	33 (8.7)	442 (8.1)
≥3 hospital stays	140 (13.0)	577 (57.0)	2297 (21.2)	96 (11.5)	181 (27.4)	1218 (15.7)	65 (9.6)	70 (18.4)	837 (15.3)

### Utilization of hospital care and outpatient care over time

**Figure 1:**
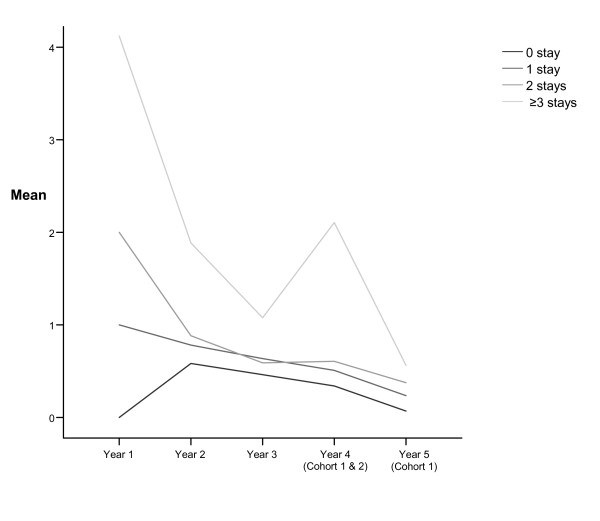
**Mean number of hospital stays in the 5 years following decision about initiating continuing care in the groups with 0, 1, 2 or ≥3 hospital stays in the first year**.

For those with 1, 2 or ≥3 hospital stays in the first year; the mean number of stays decreased significantly in the following two years but increased in the second year for those with no hospital stays in the first year (Table 3). Figure 1 shows a distinct downward slope in hospitalization rates for the second and third years in those with 3 or more hospital stays in the first year with a sharp peak in the fourth year. This figure also shows that the four groups tend to retain their starting positions in the years following with those with 3 or more stays in the first year having the highest rates and those with no hospital stays the first year having the lowest rates in the subsequent years.

**Table 3:** Mean number of hospital stays in the 3 years following the decision to initiate continuing care in the groups with 0, 1, 2 or ≥3 hospital stays in the first year

Group	First year	Second year	Third year	*p*-value^a^	Post-hoc analyses ^b^
0 hospital stay Mean (SD) (*n *= 437)	0.0 (0.0)	0.5 (1.0)	0.5 (1.1)	< 0.001	A, B
1 hospital stay Mean (SD) (*n *= 118)	1.0 (0.0)	0.8 (1.3)	0.6 (1.2)	0.009	B
2 hospital stays Mean (SD) (*n *= 56)	2.0 (0.0)	0.8 (1.5)	0.6 (1.1)	< 0.001	A, B
≥3 hospital stays Mean (SD) (*n *= 65)	4.4 (2.2)	2.0 (2.5)	1.1 (2.3)	< 0.001	A, B, C

Those who died were excluded from the analysis

^a) ^Repeated measures (with Huyunh- Feldts correction)

the Bonferroni method was used in the post-hoc analyses.

^b) ^Significant differences between: A = Years 1 and 2, B = Years 1 and 3, C = Years 2 and 3

**Figure 2:**
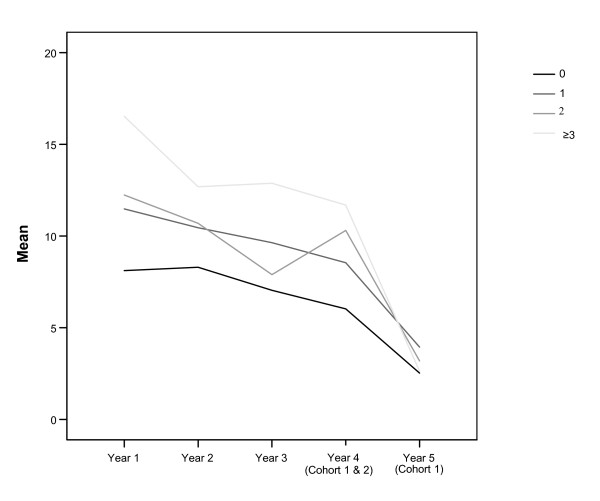
**Mean number of contacts with physicians in outpatient care in the 5 years following the decision about initiating continuing care in the groups with 0, 1, 2 or ≥3 hospital stays in the first year**.

The mean number of contacts with physicians in outpatient care decreased significantly over time in all four groups (Table 4). Figure 2 shows a downward trend in utilization of outpatient care over time in all four groups, with the group with three or more hospital stays in the first year having the highest rates and those with no hospital admissions in the first year having the lowest rates over time.

**Table 4:** Mean number of contacts with physicians in outpatient care in the 3 years following the decision to initiate continuing care in the groups with 0, 1, 2 or ≥3 hospital stays in the first year

Group	First year	Second year	Third year	*p*-value^a^	Post-hoc analyses^b^
0 hospital stay (*n *= 437) Mean (SD)	8.7 (9.2)	8.5 (8.7)	7.1 (8.3)	< 0.001	B, C
1 hospital stay (*n *= 118) Mean (SD)	13.0 (9.1)	10.9 (10.1)	9.6 (13.0)	0.005	A, B
2 hospital stays (*n *= 56) Mean (SD)	13.2 (6.5)	11.0 (7.9)	7.9 (6.3)	< 0.001	A, B, C
≥ 3 hospital stays (*n *= 65) Mean (SD)	18.6 (12.6)	14.4 (10.8)	12.9 (13.5)	0.011	A, B, C

Those who died were excluded from the analysis

^a) ^Repeated measures (with Huyunh- Feldts correction)

The Bonferroni method was used in the post-hoc analyses.

^b) ^Significant differences between: A = Years 1 and 2, B = Years 1 and 3, C = Year 2 and 3

### Comparison of those included in the repeated measure analyses and those who died

Four hundred and three people (37%) died during the three years under study and were thus not included in the repeated measure analyses. Compared to those who were included in these analyses, those who died had a significantly (p < 0.001) higher mean number of hospital stays in the first year ((mean: 1.2 (SD = 1.6) compared to mean: 0.8 (SD = 1.5)) and in the second year ((p = 0.01) mean:1.1 (SD = 1.5) compared to mean: 0.7 (SD = 1.4)) but a significantly (P = 0.04) lower mean number of contacts with physicians in outpatient care in the second year ((mean: 8.0 (SD = 7.9) compared to mean: 9.7 (SD = 9.3)). No significant differences were found regarding utilization of outpatient care in the first year.

## Discussion

A rather small proportion (13%) of those who received a decision about the initiation of continuing care was hospitalized 3 times or more during the first year. This group of high users accounted for as much as 57% of hospital stays and 21% of contacts with physicians in outpatient care for that year. This group would probably benefit the most from preventive interventions. Even though the rate of hospital and outpatient clinic utilization decreased over time in this group, it continued to have the highest rate of hospital stays and contacts in outpatient care in the two following years. The risk of someone in this group also being frequently hospitalized in the second year was 27% and the risk in the third year was 12%. This is probably an expression of poor health and a need for continued medical attention that cannot be met within continuing care or home nursing care. Apart from a more frequent hospital and outpatient clinic utilization in this group, it also had a higher proportion of men, living at home and suffering from pain and slow healing wounds and a smaller proportion with cognitive impairment than those with no hospital stays.

The data in this study were partly collected through the care and services part of the GAS Study, in which there is an unknown number of dropouts. Since there are no registers within the municipalities of those who receive a decision about continuing care, the actual number cannot be controlled for. A dropout analysis was performed for those who declined participation in 2001 which showed an over-representation of people living in special accommodation [18], indicating that those included are those with the greatest care needs but perhaps not those with the largest healthcare utilization. The unknown number of dropouts constitutes a threat to external validity of this study. Data in GAS are collected by means of a form completed by staff and the quality of data thus depends on knowledge among staff. Karlsson et al [19] (n = 152) compared the assessments made by staff in the GAS Study with the older person's views of their own degrees of dizziness, pain, anxiety and depressed mood and showed that there was poor agreement in that the older person tended to report more complaints than the personnel. Thus, there is a risk of underestimation of the degree and the proportion of health complaints. Insufficient knowledge among staff may also explain the rather high number of missing values and "unknown" responses for questions regarding functional ability and health complaints. Due to the high number of missing values and the risk of underestimation the result regarding health complaints and functional ability should be interpreted with caution.

The PASiS and PrivaStat registers were used in the collection of data. These registers form the basis for budgeting and reimbursement in the region of Skåne and can be regarded as reliable when it comes to the data used in this study i.e. the number of hospital stays and the number of contacts with physicians in outpatient care per individual. The registers allow patients to be tracked from one hospital or outpatient care facility to another within the Skåne region. Thus, the risk of both overestimation and underestimation of hospital and outpatient clinic utilization is small.

The results are in line with previous research showing that a small proportion of the population accounts for a large amount of healthcare usage during one year, looking at hospital care [1], outpatient care [20] or a combination of nursing home and hospital care usage [11]. However, this study adds valuable knowledge in that it was conducted among older people during the time following a decision about the initiation of continuing care. The study shows that despite access to continuing care those who were high users of hospital care in the first year remained as high users of both hospital and outpatient care in the subsequent years. Apparently these people are in need of more continuous medical attention and treatment which may be hard to satisfy within continuing municipal care as it is organised and provided in Sweden today. The chances of preventing frequent hospital and outpatient clinic utilization, especially frequent hospital admissions, within continuing care would perhaps increase if access to medically competent staff were improved. Several studies have shown that the increase in the number of very frail, older people within continuing care has meant an increased workload for the staff [21,22] and that staff involved in the care of older people have insufficient knowledge in such areas as medication, pressure ulcers, palliative care, dementia and nutrition [6]. This in turn places district nurses in the difficult position of having to leave medically ill patients under the supervision of unqualified staff, necessitating referrals to hospital [7]. Kayser-Jones et al [23] showed that insufficient access to adequately skilled staff in nursing homes contributed to referrals of older people to hospital. The vast majority (77%) of those who received a decision about continuing care were hospitalized at least once during the study period and everyone (except for one person) had been in contact with physicians in outpatient care. The mean number of contacts with physicians in outpatient care remained above 7 in all groups for all three years. This indicates a general need of medical healthcare beyond home nursing care. An increased availability of medically skilled staff, and perhaps also of physicians, in the municipalities would probably benefit all those who receive continuing care and perhaps serve to check frequent admissions to hospital and contacts with outpatient care.

The rate of hospital and outpatient clinic utilization decreased significantly over time in all groups, which is remarkable considering the high ages and the high mortality rates in this sample. This may be a result of the introduction of continuing care and thus a better continuity of care in general. Kristensson et al [24] showed that the utilization of medical healthcare increased in the 5 months prior to a decision about initiating continuing care. However, those who live in special accommodation and those who receive care at home can be expected to differ in several important aspects regarding their need and use of medical healthcare. This cannot be shown in this study since no distinction was made between these groups in the analyses. Several studies have previously shown that the utilization rate of, particularly, hospital care tends to decrease among older people in the time following entry into a nursing home [25,26] which may partly explain the general decrease in utilization rates over time shown in this study. The reduced utilization of hospital care over time may also be explained by a decreased access to advanced medical healthcare with increased age. Levinsky et al. [27] conducted a study on medical expenditures during the last year of life among people aged 65 year or older (n = 53 195) and found that this decreased with age, most evidently among those aged 85 years or older. Reductions in the cost of hospital care accounted for 80% of the decreased expenditures with age, due largely to less use of advanced medical healthcare, such as care in intensive care units, dialysis and use of ventilators. The decreased utilization of outpatient care over time is, however, more difficult to explain. This study is unable to show the actual reasons for a decreased hospital and outpatient clinic utilization over time. This calls for further research, especially regarding the role of primary healthcare.

The high mortality rate seen among those who received a decision about continuing care needs to be taken into consideration when investigating rates of medical healthcare utilization over time, especially if used as an outcome measures in interventional studies. Almost half the sample in this study had died 3 years after inclusion and those who died were shown to have significantly higher hospitalization rates in the first and the second years than those who were alive throughout the study period. Thus, if mortality is not taken into consideration the decreased utilization rate over time due to people dying may be misinterpreted as a positive result following intervention. Librero et al [28] reached this conclusion earlier. The greater utilization of hospital care among those who died may be a result of a more intense utilization of hospital care in the time prior to death as has been demonstrated in previous research [29,30]. The high mortality rate demonstrates the vulnerability of those who receive continuing care and points to the significance of access to palliative care in the municipalities.

The results of this study indicates that men, those who live at home, who suffer from pain or slow-healing wounds and those who are not cognitively impaired are at high risk of frequent hospital and outpatient clinic utilization. These factors may be useful in the identification of those in need of preventive interventions. Probably "at home" is a key factor, since older people who receive continuing care at home have been shown to be at higher risk of hospital readmission than those living in special accommodation, from a one year perspective [31,32] as well as a longitudinal perspective [26]. One explanation for this may be that continuity and quality of care is harder to achieve at home than in special accommodation and that those at home are more often left to follow their own or their relative's judgements in deciding about when to seek medical healthcare. Cognitive impairment is also more common among those cared for in special accommodation than among those cared for at home [18]. However, the opposite interpretation of these results could be that women, people living in special accommodation and who are cognitively impaired do not have the same access to medical healthcare as those who are less dependent and live at home. More research is needed about differences in terms of access to medical healthcare among older people receiving continuing care at home or in special accommodation.

## Conclusions

Those who have a history as high users of hospital care are at great risk of remaining as high users of both hospital and outpatient care in the subsequent years. The need for interventions targeted on preventing frequent hospital and outpatient clinic utilization in this group is thus warranted. If the rate of medical healthcare utilization is to serve as an indicator of quality of care, the actual causes or outcomes of hospital stays or with outpatient care need to be investigated [33]. This was not done in the present study, which limits the conclusions that can be drawn about quality of care. However, up to 13 hospital stays in combination with, in mean, 8 contacts with physicians in outpatient care during one year among people who receive continuing care seems worthy of note. Obviously this is a vulnerable group in need of continuous medical attention and treatment, which may be difficult to achieve within continuing care or home nursing care as it is currently provided and organized in Sweden. It might well be that increased availability of medically skilled staff in the day-to- day care of these people in the municipalities would better meet their medical needs and thus counteract their frequent hospital and outpatient clinic utilization. This is, however, a delicate problem that needs to be investigated further, especially in relation to quality of continuing care and home nursing care provided to older people at home or in special accommodation in relation to their needs.

## Competing interests

The authors declare that they have no competing interests.

## Authors' contributions

AC was involved in developing the research questions, data collection and analysis and has drafted the manuscript. UJ was involved in developing the research questions, data collection and contributed with statistical expertise in the analysis process and revised the manuscript. IRH participated in developing the GAS Study design and data collection and the design of this study, developed the research questions and supervised the realization of the study and revised the manuscript critically for research questions and important intellectual content. All authors read and approved the final manuscript.

## Pre-publication history

The pre-publication history for this paper can be accessed here:

http://www.biomedcentral.com/1472-6963/11/136/prepub
